# Clinicians’ and Advanced Cancer Patients’ Estimates of Treatment Efficacy and Toxicity in Oncologic Treatment

**DOI:** 10.3390/healthcare11152222

**Published:** 2023-08-07

**Authors:** Eun Mi Lee, Paula Jiménez-Fonseca, Alberto Carmona-Bayonas, Raquel Hernández, Patricia Cruz-Castellanos, Berta Obispo, Mónica Antoñanzas-Basa, María Palacín-Lois, Oscar A. Castillo-Trujillo, Caterina Calderon

**Affiliations:** 1 Faculty of Psychology, University of Barcelona, 08007 Barcelona, Spain; 2 Department of Medical Oncology, Hospital Universitario Central de Asturias, Instituto de Investigación del Principado de Asturias, ISPA, 33007 Oviedo, Spain; 3 Department of Medical Oncology, Hospital General Universitario Morales Meseguer, University of Murcia, 30008 Murcia, Spain; 4 Department of Medical Oncology, Hospital Universitario de Canarias, 38320 Tenerife, Spain; 5 Department of Medical Oncology, Hospital General Universitario de Ciudad Real, 13005 Madrid, Spain; 6 Department of Medical Oncology, Hospital Universitario Infanta Leonor, 28031 Madrid, Spain; 7 Department of Medical Oncology, Hospital Clínico Sant Carlos, 28040 Madrid, Spain

**Keywords:** treatment efficacy, toxicity, quality of life, curability, side effects, therapeutic communication

## Abstract

The purpose of the study was to compare curability expectations between clinicians and patients and examine the influence of sociodemographic and clinical variables on these expectations and satisfaction within the clinician-patient relationship. This prospective study, conducted from February 2020 to May 2023, involved 986 advanced cancer patients. The patients completed questionnaires assessing treatment efficacy and toxicity predictions and the Scale to Assess the Therapeutic Relationship (STAR). Seventy-four percent of advanced cancer patients had an inaccurate perception of treatment curability. Clinicians perceived male patients with lung or digestive cancer without adenocarcinoma at locally advanced stages, with fewer comorbidities and better functional status (ECOG), as having higher curability expectations. Clinicians tended to have more realistic expectations than patients, since they had to consider the presence of treatment’s side effects, while patients underestimated the possibility of experiencing these adverse effects. Patients who had more favorable expectations regarding survival and quality of life were found to be more satisfied with the care provided by their oncologists. It is crucial for patients to understand the treatment goals and establish realistic expectations in order to actively participate in decision-making and achieve a better quality of life at the end of life.

## 1. Introduction

The perception of curability is a crucial factor in the management of advanced cancer [1]. Previous studies have shown that oncologists tend to be more realistic in their assessment of curability [2,3,4], considering relevant clinical and scientific factors, while patients with advanced cancer may have unrealistic hopes and expectations regarding the possibility of a cure [5,6]. While oncologists rely on their clinical experience and the available scientific evidence to evaluate the potential cure, patients may have higher expectations of a cure, which could have been influenced by non-medical information and success stories of treatment [2,3]. These differences in expectation can lead to mental and emotional strains, including difficulties in clinician-patient communication, which can cause severe problems in shared decision-making during treatment [7,8].

The communication between oncologists and patients plays a crucial role in how curability is perceived [9,10]. Oncologists must provide clear and accurate information regarding diagnosis, prognosis, and treatment options, while patients need to understand the given information from their oncologists and adhere to the opportunity to express their concerns and doubts about the treatment [10,11]. For a better understanding of the goals of cancer treatment, it is essential to ensure informed consent and facilitate medical decision-making that aligns with the individual needs of each patient [9,12]. Although, communicating a prognosis can be distressing [13,14], effective communication from clinicians to patients can encourage patients to integrate realistic information about the possibility of a cure without losing hope, while maintaining their psychological well-being at the same time [8,15,16].

In the context of patients with advanced cancer, the importance of communication, patient-centered care, and shared decision-making is crucial [17]. These fundamental pillars foster a relationship of trust between healthcare professionals and patients by providing detailed information about diagnosis and treatment options and tailoring care to address individual needs and preferences [17,18]. In oncology, where cancer can be potentially life-threatening, trust becomes even more critical [19]. Cancer patients must navigate complex medical information and decisions while facing uncertain prognoses, radical treatments, and sometimes limited hopes for recovery [19,20]. Shared decision-making allows patients to express their values and priorities regarding their medical care, leading to decisions that align with their goals and greater satisfaction and adherence to treatment [18,20].

Despite the benefits of discussing diagnosis, prognosis, and treatment options to increase awareness of treatment intent, a significant proportion of patients, ranging from 12% to 91%, experienced misunderstandings about the severity of their disease and the purpose of cancer treatment [7,21,22]. A landmark study conducted by Weeks et al. (2012) revealed that most newly diagnosed patients with metastatic lung or colorectal cancer believed they could be cured through chemotherapy [21]. These unrealistic expectations regarding curability may originate from different sources, each of which contributes to the phenomenon in a distinct manner. These sources include ineffective communication, which can lead to misunderstandings and false hopes; patient denial of the incurable nature of the disease, wherein individuals may find it challenging to accept the reality of their condition; and the use of optimism as a coping strategy, which may foster a hopeful outlook despite the challenging circumstances [6,23,24].

Scientific evidence indicates that a significant number of patients in palliative care believe that their therapy aims for a cure but are unaware of their life expectancy [21,25,26]. Patients with advanced malignant neoplasms often overestimate the benefits of chemotherapy and hold misconceptions about the therapeutic intent of treatment [7,9,27]. Female patients and those with secondary or higher education are more likely to understand their diagnosis [28]. However, patients with lower education levels tend to lack awareness of treatment intent [29,30]. Additionally, older patients with lower incomes and a lack of social support tend to have misconceptions about treatment goals [6,31]. It is important to note that misconceptions about treatment goals are not limited to patients in palliative care, as evidenced by a study that included patients with early-stage solid malignant tumors [31,32]. Differences in the perception of curability also influence treatment decisions [33]. Differences in the perception of curability also influence treatment decisions [29]. Patients may opt for more aggressive treatments in pursuit of a potential cure, while oncologists may recommend treatment options focused on symptom control and improving quality of life.

In the context of advanced cancer, discrepancies in the perception of curability between oncologists and patients pose challenges in communication and shared decision-making. Understanding these differences and their impact can enhance the quality of care. The objectives of the current study were to compare expectations of curability between clinicians and patients with advanced cancer and to assess whether sociodemographic and clinical variables, such as the age of the clinician and patient and years of experience, influence the expectations and satisfaction of the received treatment. We hypothesize that clinicians will have more optimistic expectations of curability compared to patients with advanced cancer. Additionally, sociodemographic and clinical variables, such as the age of clinicians and patients and the years of experience of the clinicians, will influence treatment expectations and satisfaction. Patient satisfaction with the treatment received will be positively associated with the alignment of expectations with their clinicians.

## 2. Materials and Methods

### 2.1. Study Design and Population

This research, characterized by its prospective and cross-sectional design, took place from February 2020 through May 2023 within various medical departments across Spanish hospitals. Within the scope of Spain’s 17 autonomous communities, there are a total of 210 departments specializing in medical oncology. The current study was conducted within an estimated 7.1% of these medical oncology departments, distributed among nine of the autonomous communities. These locations served as the primary sites for comprehensive data collection and analysis. Furthermore, this geographical distribution was intentionally selected to ensure a broad representation of all regional zones. The objective of the study was to gather data on advanced cancer patients who were not suitable for curative therapy. Patients were enrolled during their initial visit to the medical oncologist, where they were informed about the diagnosis, disease stage, and available systemic antineoplastic treatments. The criteria for participant eligibility necessitated that the individual be at least 18 years of age and possess a histopathological diagnosis of advanced cancer while concurrently being ineligible for surgical intervention or other therapeutic curative measures. Candidates were disqualified for inclusion based on a variety of factors. These encompassed any physical status, age, or comorbidity deemed incompatible with antineoplastic treatment as per the supervising oncologist’s discretion, prior treatment for a distinct advanced cancer within the preceding two-year period, and any pre-existing medical, sociological, familial, or personal circumstances that could potentially obstruct their participation. Patients with cognitive impairment, severe deterioration of general status, or an inability to comprehend or respond to questionnaires were also excluded. The research activities secured an official endorsement from the Ethics Review Committee associated with each participating institution and also gained approval from the Spanish Agency of Medicines and Health Products (AEMPS), as denoted by the assigned identification code: ES14042015. This dual-level authorization process ensured that the study adhered to the ethical standards upheld both by the institutions involved and the national health regulatory authority. Informed consent was obtained from all participants. Data collection involved completing questionnaires and extracting clinical information from interviews and medical records of each participating patient. The process was standardized across all participating hospitals, and patient data was obtained from their respective treatment institutions. Participation was voluntary and anonymous, with no impact on patient care. Individuals who consented to partake in the study proceeded to affix their signatures on the consent form, after which they were provided with comprehensive guidance on how to accurately complete the written questionnaires. These documents were filled out at the participant’s residence and returned to the auxiliary staff during their subsequent scheduled visit. Medical oncologists utilized a web-based platform (www.neoetic.es) to update and collect data.

### 2.2. Description of Variables

A standard self-report form was employed to gather the sociodemographic attributes of the participants. The collection of data regarding cancer and its treatment was executed by the oncologist, who utilized both direct patient interviews and a comprehensive review of their medical histories. Subsequently, each patient’s functional status was categorized by the oncologist utilizing the Eastern Cooperative Oncology Group Performance Status (ECOG-PS) scale, which operates on a spectrum from 0 (signifying asymptomatic) to five (indicating deceased). It was mandated that any ECOG score could be considered valid, provided the oncologist found the patient to be an apt candidate for systemic therapy. The oncologist dispensed the questionnaire during the patient’s consultation, where the patient was informed about systemic antineoplastic treatment, allowing them to complete the questionnaire in the comfort of their own home prior to commencing the treatment.

The prediction of treatment efficacy and toxicity were obtained from the oncologist who attended the patient’s first visit to assess the suitability of administering oncological treatment. This information was collected using a standardized questionnaire with four questions [3,34]. Oncologists were asked to indicate, on a numerical scale from 0 to 100, whether the treatment would help in curing the disease, improve quality of life, alleviate symptoms, or reduce the risk of severe side effects. Clinicians were also asked to provide data about themselves, which included gender, age, professional degree or education level, and years of experience. Patients were asked to answer the same four questions regarding whether they believed the treatment would help cure their cancer, improve their quality of life, alleviate symptoms, and foresee experiencing severe side effects, on a numerical scale from 0 to 100.

Conceived by McGuire-Snieckus and colleagues in 2007, the Scale to Assess the Therapeutic Relationship (STAR) functions as an instrument for gauging the relationship dynamics between patients and healthcare providers within the framework of community mental health care environments. The STAR tool presents in two distinct forms: a patient-oriented version, denoted as STAR-P, and a clinician-focused variant, labeled as STAR-C. Each of these versions incorporates a set of 12 components, assessed using a five-point Likert scale where the responses span a range from 0 to 4.

The STAR-P is completed by patients to assess their therapeutic relationship with the clinician, while the STAR-C is completed by clinicians to evaluate their therapeutic relationship with the patient. Each version includes three subscales. The STAR-P subscales are positive collaboration, positive clinician input, and non-supportive clinician input. The STAR-C subscales are positive collaboration, emotional difficulties, and positive clinician input. The original versions of both STAR-P and STAR-C demonstrated acceptable internal consistency, test-retest reliability, and factorial validity [35].

### 2.3. Statistical Analyses

Descriptive statistics were included for patients’ and clinicians’ demographic information as well as patients’ clinical data. In order to verify the differences between clinicians’ and patients’ predictions of treatment efficacy and toxicity, *t*-tests were executed for quantitative variables and chi-square tests for qualitative variables. To confirm the differences in the prediction of treatment efficacy and toxicity between clinicians and patients based on their age, an ANOVA was used. Bonferroni correction was used for post-hoc contrast. Eta squared (η^2^) was applied to assess the effect size of continuous variables. Eta-squared ranges between 0 and 1, with η^2^ ~ 0.01 for a small, η^2^ ~ 0.06 for a medium, and η^2^ > 0.14 for a large effect size. Pearson correlation coefficients were calculated to examine the relationship between the prediction of treatment efficacy and toxicity and demographic variables, as well as the therapeutic relationship between patients and clinicians using questionnaires. A statistical significance level of 0.05 was established. The statistical analyses were performed using the IBM-SPSS software package for Windows, version 26.0 (SPSS, Inc., Armonk, NY, USA).

## 3. Results

### 3.1. Participants

The involved professionals gathered a group of 1053 participants, of whom 67 were subsequently disqualified from the study. The reasons for disqualification varied: 20 subjects failed to meet the established inclusion criteria, 19 encountered an exclusion criterion, and 28 were eliminated due to data insufficiency. This led to a final participant count of 986 individuals. Among this group, 45.4% identified as female, with an average age of 65.5 years spanning from 35 to 90 years old. A significant portion, approximately 67.2%, were in a marital or partnered relationship, possessed an elementary level of education (54%), and were retired or unemployed (49.6%). The most common histology and metastases shown in patients were Adenocarcinomas (64.6%) and cancers in stage IV (79.9%). Among treatment modalities, the majority were treated with chemotherapy (54.4%). Consecutively by immunotherapy (6.6%) and antidiana (5.8%). Estimated survival was less than 18 months in 44.5% of the sample (Table 1).

Oncologists believed that men had a higher expectation of cure than women (M = 39.2 vs. M = 34.7, F = 7.532, *p* = 0.006, η^2^ = 0.008), as well as patients with lung or digestive cancer (F = 13.517, *p* = 0.001, η^2^ = 0.054), those who do not have adenocarcinoma (M = 35.8 vs. M = 39.3, F = 4.495, *p* = 0.034, η^2^ = 0.005), those with locally advanced stage (M = 47.7 vs. M = 34.3, F = 41.910, *p* = 0.001, η^2^ = 0.043), and better ECOG (M = 43.8 vs. M = 32.9, F = 43.697, *p* = 0.001, η^2^ = 0.044).

Regarding patients, men also believed they had a higher expectation of cure compared to women (M = 78.9 vs. M = 70.4, t = 3.733, *p* = 0.001, η^2^ = 0.015), as well as patients with lung or digestive cancer (F = 13.343, *p* = 0.001, η^2^ = 0.054), and those with better ECOG (M = 80.1 vs. M = 72.0, F = 12.138, *p* = 0.001, η^2^ = 0.013).

### 3.2. Treatment Expectations

The opinions on treatment expectations from both clinicians and patients can be seen in Table 2. The clinician had a lower expectation of cure than the patient in the case of oncological treatment (74% versus 13%, respectively; X^2^ = 32.803, *p* = 0.008), but expected a better quality of life with the treatment (89% versus 80% of patients, respectively; X^2^ = 33.603, *p* = 0.001). Clinicians had higher expectations of experiencing side effects (79% versus 60% of patients; X^2^ = 34.768, *p* = 0.004). Regarding symptom relief, both clinicians and patients expected favorable results (90% versus 82%, respectively) without significant differences between them.

### 3.3. Factors Affecting Prediction

There were no significant differences in the assessment of healing and side effect expectations between male and female oncologists. However, male oncologists had higher expectations of patients living better (90 vs. 84, respectively; t = 4.704, *p* = 0.001), relieving cancer symptoms (90 vs. 84, respectively; t = 4.320, *p* = 0.001), and experiencing more side effects (51 vs. 46, respectively; t = 1, *p* = 0.024) than their female counterparts.

As for the patients, there were differences between men and women in their treatment expectations of healing (78 vs. 70, respectively; t = 3.733, *p* = 0.001), living better (85 vs. 81, respectively; t = 2.022, *p* = 0.043), relieving cancer symptoms (83 vs. 79, respectively; t = 1.979, *p* = 0.048), and experiencing more side effects (51 vs. 46, respectively; t = −2.529, *p* = 0.012). Men believed that cancer treatment could cure them, improve their quality of life, and relieve their symptoms more than women. On the contrary, women reported experiencing more side effects than men.

Clinicians’ age and years of experience were negatively correlated with expectations of healing and relieving cancer symptoms. Younger clinicians were more likely to have patients with high expectations, while older and more experienced clinicians were less likely.

Treatment expectations in patients were not related to the patient’s age, the clinician’s age, or their years of experience but were related to satisfaction with the doctor-patient relationship. Patients with expectations of cure, improved quality of life, and symptom relief were more satisfied with their oncologist (Table 3).

## 4. Discussion

The following research was important to conduct since it was the first multicenter study in Spain that assessed the perception of curability in patients with advanced cancer. The results showed that 74% of patients with advanced cancer had an inaccurate perception of curability regarding treatment. This misperception was also found in 69–91% of metastatic patients who believed in the curative intent of their treatment [7,21,22]. Additionally, misconceptions of treatment goals by patients can be explained by coping strategies such as denial, which were well described in this patient population [36,37]. Overall, patients with advanced cancer often have overly optimistic expectations regarding their chances of survival and cure rates [3,5,34,38]. It is vital to understand treatment goals for patients so they can settle on more suitable decision-making aligned with their preferences and also be able to establish realistic expectations about treatment outcomes. Patients who understand that their disease is incurable are more likely to receive palliative care and have a better quality of life until its end [2].

In the current study, findings revealed that male patients with non-adenocarcinoma lung or digestive cancer, locally advanced stages, and better ECOG performance status were perceived by oncologists to have higher expectations of cure. Male patients also had higher expectations of cure than female patients, especially those with lung or digestive cancer and better functional status. These gender differences in expectations of cure align with existing research in psychosocial oncology. A study conducted by Sharma et al. (2015) on patients with advanced cancer found that female patients reported higher rates of palliative care discussions with their clinicians, were less likely to receive aggressive treatment in the last two weeks of life, and were more likely to receive palliative care compared to men [39]. This suggests that female patients may be more open to discussing palliative care options due to a potentially lower expectation of curability. Furthermore, other studies have shown that colon cancer patients had higher expectations of curability than lung cancer patients [6,24,40]. It has been observed that these female patients and patients with higher education also had a more accurate perception in terms of the curability of their disease [4,29,30]. This suggests that their level of knowledge and understanding of their medical condition allows them to have a more realistic view of treatment options and the possibilities of recovery. In the present study, functional impairments were associated with lower expectations of cure in both patients and clinicians. This result was expected since functional impairments are common in patients with advanced cancer [41,42], which can hinder the understanding of the actual situation and treatment options. Functional impairments, including problems with memory, concentration, fatigue, and physical and/or emotional limitations, can pose challenges in comprehending the true situation and treatment choices, which can potentially lead to an inaccurate interpretation of cancer’s curable prognosis. Moreover, these limitations can significantly impact patients’ daily lives, hindering their ability to process information and effectively communicate with healthcare professionals. Further studies will be needed to analyze if these differences in expectations persist throughout the process. It is essential for clinicians to be aware of these differences to address them and grasp a better understanding of them, ultimately improving the quality of care by satisfying the needs and expectations of each patient. In order to optimize patient-centered care, it is essential for clinicians to be aware of gender-related differences and functional impairment expectations. A better understanding of psychosocial dynamics can improve the quality of care by addressing the unique needs and expectations of each patient throughout their cancer journey.

Clinicians and patients have different expectations regarding the occurrence of treatment side effects. Clinicians tend to have more realistic expectations regarding the presence of treatment-related adverse effects, while patients may underestimate the possibility of experiencing these side effects. However, both clinicians and patients expect symptom relief and improvement with treatment. Consistent with other research, cancer patients often have different treatment expectations compared to their clinicians [6,43]. As such, patients with less realistic expectations tend to experience higher psychological distress [3,44] and a poorer quality of life [10]. On the contrary, patients with more realistic survival expectations were more satisfied with the care provided by their clinicians [10]. Improving prognostic awareness can help patients hope for the best but prepare them for the worst [45], which can prevent problematic consequences due to a lack of prognostic knowledge [22,46].

In the study, findings indicated that younger clinicians were more likely to have high expectations of cure and symptom relief for their cancer patients, while older and more experienced clinicians were less likely to have high expectations. Consistent with other research, it has been found that cancer patients treated by younger oncologists had higher survival rates compared to patients treated by older oncologists [47]. The authors speculated that this may be due to younger oncologists being more likely to follow the latest clinical practices and apply more aggressive treatments than older oncologists [47]. Conversely, cancer patients treated by more experienced clinicians had a lower likelihood of being hospitalized in the final days of life compared to patients treated by less experienced clinicians [48,49], which suggested that more veteran clinicians are better at anticipating and managing end-of-life symptoms. It is important for clinicians to be able to communicate the limitations of treatments in a more prepared and clear way, since more experienced clinicians may be better at addressing end-of-life symptoms than younger clinicians, which is often seen in patients with advanced cancer.

Furthermore, patients who were more satisfied with the care provided by their oncologists were those who had higher expectations in terms of survival and quality of life. Consistent with these findings, there were studies indicating that patients with high expectations and hopes that the treatment would work were more satisfied with the care received from their oncologists [50,51,52]. In contrast, other researchers suggested that if patients experienced unrealistic expectations of a cancer cure, they would tend to display a lower quality of life and higher psychological distress [3,34], due to unrealistic expectations that can lead to greater disappointment and frustration when desired treatment results are not achieved. Understanding and addressing patients’ expectations can improve the quality of care and patient satisfaction.

The limitations found in the study were, first, that it was based on the subjective perception of patients and clinicians, which could be influenced by their own expectations and biases. Second, although it is a multicenter study in Spain, the results may not be generalizable to other populations or cultures. Third, while the sample size was relatively large, imbalances in the distribution of patients in terms of age, gender, cancer type, and disease stage could have subsisted, hindering the generalization of the results. Finally, the study did not consider the influence of socioeconomic and cultural factors that could impact patients’ perceptions of cancer curability.

## 5. Conclusions

In conclusion, the study showed that many patients with advanced cancer had an inaccurate perception of their curability, which could lead to unrealistic expectations and inappropriate treatment decisions. It is crucial for patients to understand the treatment goals and establish realistic expectations in order to participate in decision-making and have a better quality of life at the end of their lives. Clinicians must be aware of the differences between their expectations and those of their patients, address them properly, and fully understand them to improve the quality of care and meet the patients’ needs. Moreover, improving prognostic awareness may help patients expect the best but prepare for the worst, which could prevent the problematic consequences of a lack of prognostic knowledge.

Future research should prioritize interventions that promote shared decision-making in advanced cancer patients, empowering them to actively participate in treatment options [17,18]. This includes providing communication skills training for healthcare professionals. This suggests that understanding the impact of cultural and psychosocial factors on decision-making is crucial for improving patient outcomes along the cancer journey.

## Figures and Tables

**Table 1:** Baseline clinical-psychosocial characteristics of patients (n = 986).

Characteristics	n	%	Clinician ^a^ *p* Values	Patients ^b^ *p* Values
Gender (female)	448	45.4	0.001	0.001
Age (≥65)	588	59.6	--	--
Marital status (married or partnered)	663	67.2	--	--
Education (primary level)	533	54.0	--	--
Work (retired)	490	49.6	--	--
Cancer type				
Bronchopulmonary	292	29.6	0.001	0.001
Digestive	210	21.3		
Pancreas	91	9.2		
Breast	79	8.0		
Others	314	31.8		
Histology			0.035	--
Adenocarcinoma	637	64.6		
Others	349	35.4		
Metastasis			0.001	--
Advanced locally	198	20.1		
Stage IV	788	79.9		
Type of treatment			--	--
Chemotherapy	536	54.4		
Immunotherapy	65	6.6		
Antidiana	57	5.8		
Others	328	33.3		
Elixhauser comorbidities			---	--
≤4	394	40.0		
>4	592	60.0		
ECOG (0)	371	37.6	0.001	0.001

^a^ Clinicians’ expectations of healing. ^b^ Patients’ expectations of healing.

**Table 2:** Clinicians’ and Patients’ treatment expectations.

Variables	M (SD)	Do Not Know	Very Low	Low	High	Very High	*p* Value
			0–25%	26–50%	51–75%	76–100%	
Expectations of healing							0.008
Clinicians’ expectations	37 (25)	10	53	21	10	6	
Patients’ expectations	75 (34)	13	4	9	19	56	
Expectations of living better							0.001
Clinicians’ expectations	85 (25)	--	1	10	36	53	
Patients’ expectations	83 (26)	5	3	9	22	58	
Expectations of relieving cancer symptoms							---
Clinicians’ expectations	85 (17)	--	1	9	37	53	
Patients’ expectations	81 (27)	6	3	9	24	58	
Expectation of having many side effects							0.004
Clinicians’ expectations	48 (24)	4	28	48	10	9	
Patients’ expectations	51 (35)	21	13	26	21	19	

**Table 3:** Correlations between prognostic prediction and sociodemographic variables.

Variables	Clinicians’ Age	Years of Experience	Patients’ Age	STAR-C Clinician	STAR Patient
Clinicians’ expectation					
Expectations of healing	−0.178 **	−0.125 **	−0.031	−0.003	---
Expectations of living better	−0.002	−0.182	−0.055	0.065	---
Expectations of relieving cancer symptoms	−0.134 **	−0.080 *	−0.040	0.051	---
Expectation of having many side effects	−0.032	0.037	−0.061	−0.026	
Patients’ expectation					
Expectations of healing	−0.047	−0.037	−0.001	---	0.203 **
Expectations of living better	−0.010	−0.013	−0.001		0.231 **
Expectations of relieving cancer symptoms	0.051	0.042	−0.029	---	0.188 **
Expectation of having many side effects	0.002	−0.006	0.043	---	0.025

* *p* < 0.01; ** *p* < 0.001.

## Data Availability

The datasets generated and analyzed during the current study are not publicly available for reasons of privacy. They are, however, available (fully anonymized) from the corresponding author on reasonable request.
